# A risk prediction stratification for non-mass breast lesions, combining clinical characteristics and imaging features on ultrasound, mammography, and MRI

**DOI:** 10.3389/fonc.2024.1337265

**Published:** 2024-10-17

**Authors:** YaMie Xie, Xiaoxiao Zhang

**Affiliations:** ^1^ Department of CT/MRI, The Second Affiliated Hospital of Fujian Medical University, Quanzhou, China; ^2^ Department of Ultrasound, Ruijin Hospital, Shanghai Jiao Tong University School of Medicine, Shanghai, China

**Keywords:** ultrasound, mammography, MRI, non-mass BI-RADS, clinical factors

## Abstract

**Objectives:**

Given the inevitable trend of domestic imaging center mergers and the current lack of comprehensive imaging evaluation guidelines for non-mass breast lesions, we have developed a novel BI-RADS risk prediction and stratification system for non-mass breast lesions that integrates clinical characteristics with imaging features from ultrasound, mammography, and MRI, with the aim of assisting clinicians in interpreting imaging reports.

**Methods:**

This study enrolled 350 patients with non-mass breast lesions (NMLs), randomly assigning them to a training set of 245 cases (70%) and a test set of 105 cases (30%). Radiologists conducted comprehensive evaluations of the lesions using ultrasound, mammography, and MRI. Independent predictors were identified using LASSO logistic regression, and a predictive risk model was constructed using a nomogram generated with R software, with subsequent validation in both sets.

**Results:**

LASSO logistic regression identified a set of independent predictors, encompassing age, clinical palpation hardness, distribution and morphology of calcifications, peripheral blood supply as depicted by color Doppler imaging, maximum lesion diameter, patterns of internal enhancement, distribution of non-mass lesions, time–intensity curve (TIC), and apparent diffusion coefficient (ADC) values. The predictive model achieved area under the curve (AUC) values of 0.873 for the training group and 0.877 for the testing group. The model’s positive predictive values were as follows: BI-RADS 2 = 0%, BI-RADS 3 = 0%, BI-RADS 4A = 6.25%, BI-RADS 4B = 26.13%, BI-RADS 4C = 80.84%, and BI-RADS 5 = 97.33%.

**Conclusion:**

The creation of a risk-predictive BI-RADS stratification, specifically designed for non-mass breast lesions and integrating clinical and imaging data from multiple modalities, significantly enhances the precision of diagnostic categorization for these lesions.

## 1 Introduction

The magnetic resonance Breast Imaging Reporting and Data System (BI-RADS) lexicon of the American College of Radiology classifies breast lesions as mass, non-mass, and foci. Non-mass enhancement (NME) on breast magnetic resonance imaging (MRI) is characterized by distinct internal enhancement features that set it apart from the surrounding normal breast parenchyma in the absence of an associated mass, as per the BI-RADS lexicon (1).

NME on breast ultrasound (US) is characterized as a sonographic finding that is identifiable in at least two imaging planes but does not exhibit the three-dimensionality or the distinct visibility of a mass, as defined by the Radiological Society of North America (RSNA, 2023). While imaging modalities are generally adept at detecting mass lesions and facilitating accurate diagnoses, NME poses distinct clinical challenges. This is because the conventional morphological and kinetic features that are indicative of mass lesions may not be as effective for NME lesions. The presence of various benign and malignant lesions that exhibit non-specific enhancement on breast MRI may lead to an elevated false-positive rate when compared to mass lesions (2–4).

Previous research has predominantly focused on differentiating non-mass lesions (NMLs) from mass lesions, employing a range of machine-learning techniques to boost diagnostic accuracy within individual imaging modalities or to evaluate the diagnostic efficacy of specific imaging methods for NMLs (5–7). However, there has been a paucity of studies exploring the BI-RADS classification for NMLs, with some suggesting that the existing classification system has limited applicability in categorizing NMLs identified by MRI imaging (8).

Conversely, other studies have highlighted the significance of ultrasound in the diagnosis of non-mass breast lesions, proposing classification systems that rely on imaging features to interpret NMLs detected via US and to stratify their cancer risk (9–11). Despite these efforts, there remains a lack of consistency in the description of lesion characteristics across different studies (12), and to date, no standardized classification system for NMLs on ultrasound has been universally adopted (13). The advent of improved resolution in breast ultrasonography, coupled with the integration of innovative diagnostic techniques such as strain elastography and shear wave elastography, has led to a progressive enhancement in the diagnostic value of ultrasound for the assessment of non-mass lesions.

Building upon these observations, this study aims to construct a classification model based on the clinical and imaging features of NMLs on ultrasound, mammography, and breast MRI. The authors have meticulously defined the imaging features of NMLs and consolidated redundant indicators. By selecting the most sensitive indicators, a new non-mass BI-RADS risk stratification using a nomogram was created. We developed a novel risk-predicted BI-RADS stratification system for non-mass breast lesions that integrates clinical characteristics with imaging features from ultrasound, mammography, and MRI. This endeavor seeks to address the challenge of the high false-positive rate of NMLs, enhance diagnostic accuracy, and aid radiologists in effectively managing all NMLs detected in the breast.

## 2 Materials and methods

This retrospective analysis was granted approval by the Ethics Committee of Ruijin Hospital, which is affiliated with Shanghai Jiao Tong University School of Medicine, with a waiver for the requirement of written informed consent from the participants.

### 2.1 Study sample

**Figure 1:**
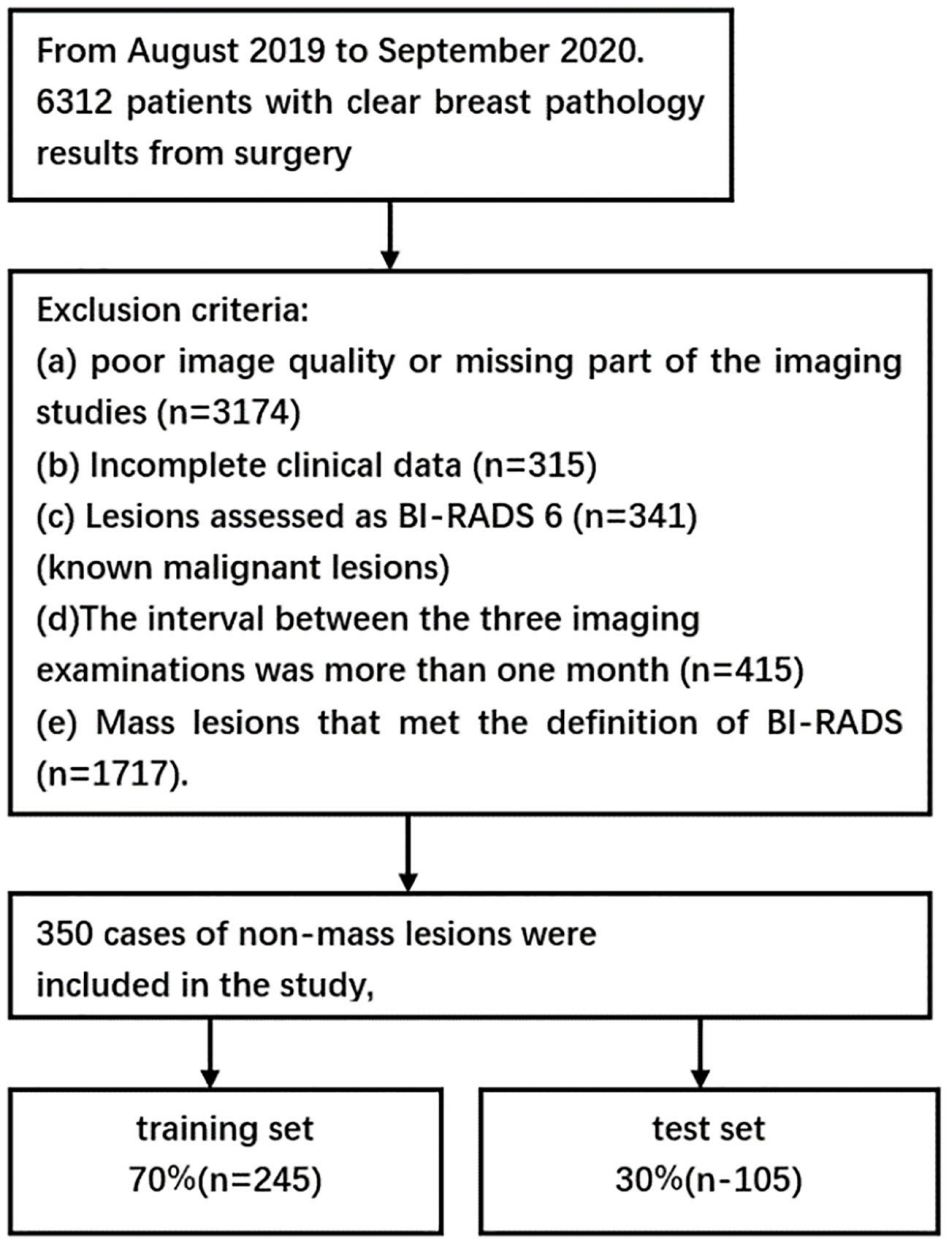
Flowchart showing the number of participants included and the reasons for exclusion from the study.

From August 2019 to September 2020, a total of 6,312 patients with final pathological results obtained via surgery or core needle biopsy were enrolled. Of these, 2,067 patients underwent the three primary imaging examinations—ultrasound, mammography, and MRI—at our hospital prior to surgery and fulfilled the inclusion criteria. This group comprised 1,717 patients with mass breast lesions and 350 patients with NMLs. The exclusion criteria were as follows: a) poor image quality or incomplete imaging examinations (*n* = 3,174); b) incomplete clinical data (*n* = 315); c) lesions classified as BI-RADS 6 (*n* = 341), indicating known malignant lesions; d) a time interval exceeding 1 month between the imaging examinations (*n* = 415); and e) mass lesions conforming to the definition in the fifth edition of BI-RADS 2013 (*n* = 1,717). Ultimately, 350 cases of NML were included in the study. All patients were women with a mean age of 52 years (age range: 19–90 years), including 117 benign cases and 233 malignant cases. A flowchart depicting the number of participants included and the reasons for exclusion is presented in **Figure 1**, while **Table 1** outlines the pathological types of the patients.

**Table 1:** The pathological types of patients.

	Group	Number (%)
	Malignant	Total *n* = 233
**Pathological type**	Invasive ductal carcinoma	34 (14.59)
	Ductal carcinoma *in situ*	86 (36.91)
	Mixed carcinoma	45 (19.31)
	Invasive lobular carcinoma	7 (3.00)
	Papillary carcinoma	22 (9.44)
	Other invasive carcinoma	39 (16.74)
	Benign	Total *n* = 117
	Fibrocystic changes	57 (48.72)
	Intraductal papilloma	13 (11.11)
	Fibroadenoma	9 (7.69)
	Adenosis+intraductal papilloma/fibroadenoma	28 (23.93)
	Adenosis with ductal dilatation	10 (8.54)

Unless otherwise indicated, the value represents the number of patients, with the percentage shown in parentheses.

### 2.2 Acquisition system

The mammography was performed using the American Hologic mammography machine: the projection positions were mainly in the MLO position and the CC position. If necessary, local compression-magnified irradiation and special body position irradiation were given.

The ultrasound examination was performed with a Mindray Resona 7 system, a linear array probe, and the frequency was 10.0 to 14.0 MHz. Ultrasound examination of the entire breast should be performed from the posterior axillary line to the parasternal line, with the nipple as the center, the nipple–areola complex area, and the axillary lymph nodes. The transducer is to be lightly supported on the skin, without pressing the skin to avoid an underestimation of the vascularization. Elastography is converted to the SE (strain elasticity) model when the longest axis view of the lesions is displayed on the 2D image, and the probe should be handled gently.

All the breast MRI examinations were performed on a 1.5-T unit (MAGNETOM Aera; Siemens Healthcare, Erlangen, Germany) with a dedicated 18-channel phased-array breast coil. The protocol included axial T1-weighted, T2-weighted fat-suppressed diffusion-weighted imaging (DWI, *b* value is 1,000 s/mm^2^), T1-weighted fat-suppressed dynamic enhancement scan: 1 stage enhancement 90 s + 5 stage enhancement (90 s × 5) after injection of 20 mL gadolinium meglumine, and then the images were uploaded to the Picture Archiving and Communication System (PACS). Post-processing included T1-weighted subtraction, T1-weighted maximum intensity projection, and subtracted sagittal reconstruction. The apparent diffusion coefficient (ADC) was measured, and the time–signal intensity curve (TIC) was obtained.

### 2.3 Image analysis

Using this methodology, images were downloaded from the hospital PACS in Digital Imaging and Communications in Medicine (DICOM) format. Two radiologists (XXZ and YMX, with 10 and 4 years of experience in breast diagnosis, respectively) reviewed the images that included ultrasound, mammography, and MRI without knowing the pathological results.

The characteristics of each lesion were described and recorded according to the fifth edition of the ACR BI-RADS lexicon and according to the associated imaging variables on ultrasound of non-mass in the literature published by Park et al. (14), and when the conclusions were different, a consensus was reached after discussion with a third radiologist (MWC, with ≥10 years of experience in breast diagnosis).

On MRI, enhancement patterns can be classified as mass, non-mass, or foci-enhancing lesions. In this study, the identification of NMLs on MRI means that the lesions do not belong to a 3D mass or have distinct features of a mass. On mammography, the identification of NMLs relies on differences in glandular density or distribution of microcalcifications due to the absence of an occupying effect. To date, there is still no standardized approach to classify and evaluate non-mass findings on US. An NME on breast US is defined as a sonographic finding that is identified in two or more imaging planes but lacks the three-dimensionality or conspicuity of a mass (RSNA, 2023). The “maximum diameter of the lesion” was taken as the largest value on MRI, and the “associated features” were only cited in the MRI and ultrasound examination, because MRI and ultrasound can better depict lesions due to their superior soft tissue contrast. At the same time, the clinical information of the patients was collected online.

### 2.4 Methods and statistical analysis

Statistical analysis was conducted using SPSS Statistics (version 26.0, USA), while R software (version 4.0.5) was employed for comprehensive data analysis. Continuous variables were expressed as mean ± standard deviation. Univariable analyses were executed with Student’s *t*-test or one-way ANOVA for normally distributed data, and the Mann–Whitney *U* test was applied for data that were not normally distributed. Categorical variables were presented as frequencies and percentages, with the chi-square test or Fisher’s exact probability analysis utilized for assessment. The LASSO logistic regression algorithm, facilitated by the glmnet R package, was implemented to diminish the feature dimension and to discern the imaging and clinical information pertinent to the differential diagnosis of benign and malignant conditions. Participants were randomly allocated into two cohorts using R software: 245 cases (70%) formed the training set, and 105 cases (30%) constituted the test set. The ROC curve analysis was conducted to appraise the diagnostic efficacy of both sets in distinguishing between benign and malignant lesions. For each predictive factor depicted in the nomogram, scores were assigned, and the aggregate score was calculated to determine the total score. Subsequently, the model was used to derive the probability of malignancy for each patient. Lesions were reassigned to categories in accordance with the positive predictive values outlined in the BI-RADS guidelines. A *p*-value of less than 0.05 was set as the threshold for statistical significance.

## 3 Results

### 3.1 Patient clinical information and imaging indicators

Of the 350 NMLs examined, 233 cases (66.6%) were identified as malignant, while 117 cases (32.4%) were benign. A detailed breakdown of these results is presented in **Table 1**. The patient cohort was entirely female, with an average age of 52 years and an age range of 19 to 90 years.

Univariate analysis revealed statistically significant differences across several parameters: the maximum diameter of the lesions, menstrual status, hardness determined by clinical palpation, distribution and morphology of calcifications, posterior echo, peripheral vascularity as indicated by color Doppler flow imaging (CDFI), strain elasticity (SE) assessed via ultrasound, internal enhancement patterns and distribution of NME, TIC, ADC values, and associated MRI features. A comprehensive presentation of these detailed results can be found in **Table 2**.

**Table 2:** Comparison of relevant clinical symptoms and imaging features of benign and malignant patients.

	Parameters	Characteristics	Malignant (%)	Benign (%)	*p*	PPV
**Clinical symptom**	**Tenderness**	No	198 (84.98)	104 (88.89)	0.316	65.6%
		Yes	35 (15.02)	13 (11.11)		72.9%
	**Clinical palpation hardness**	Hardness	180 (77.25)	60 (51.28)	<0.001	75%
		Soft	53 (22.75)	57 (48.72)		48.2%
	**Nipple discharge**	No	208 (89.27)	111 (94.87)	0.082	65.2%
		Yes	25 (10.73)	6 (5.13)		80.6%
	**Menstrual status**	Premenopausal	107 (45.92)	77 (65.81)	<0.001	58.2%
		Postmenopausal	126 (54.08)	40 (34.19)		75.9%
	**Hormone therapy**	No	213 (91.42)	108 (92.31)	0.775	66.4%
		Yes	20 (8.58)	9 (7.69)		69.0%
	**Family history of breast cancer**	No	208 (89.27)	101 (86.33)	0.419	67.3%
		Yes	25 (10.73)	16 (13.68)		61.0%
	**Previous breast surgery**	No	212 (90.99)	104 (88.89)	0.532	67.1%
		Yes	21 (9.01)	13 (11.11)		61.8%
	**Age**	40–60 years	138 (59.23)	70 (59.83)	0.038	66.3%
		<40 years	35 (15.02)	28 (23.93)		55.6%
		>60 years	60 (25.75)	19 (16.24)		75.9%
**MRI features**	**Internal enhancement patterns**	Unenhanced/homogeneous	78 (33.48)	72 (61.54)	<0.001	52%
		Heterogeneous/clumped/clustered ring	155 (66.52)	45 (38.46)		77.5%
	**Distribution**	Regional	41 (17.60)	12 (10.26)	<0.001	77.4%
		Multiple regions	11 (4.72)	3 (2.56)		78.6%
		Focal/linear	65 (27.90)	86 (73.50)		43.0%
		Diffuse	9 (3.86)	1 (0.86)		90.0%
		Segmental	107 (45.92)	15 (12.82)		87.7%
	**TIC**	Plateau/washout	137 (58.80)	29 (24.79)	<0.001	82.5%
		Unenhancement/persistent	96 (41.20)	88 (75.21)		52.2%
	**ADC, median [IQR]**		1.00 (0.88, 1.20)	1.28 (1.00, 1.48)	<0.001	
	**BPE**	Moderate/marked	16 (6.87)	13 (11.11)	0.174	55.2%
		Minimal/mild	217 (93.13)	104 (88.89)		67.6%
	**FGT**	Heterogeneous fibroglandular tissue/extreme fibroglandular tissue	129 (55.37)	73 (62.39)	0.209	63.9%
		Almost entirely fatty/scattered fibroglandular tissue	104 (44.64)	44 (37.61)		70.3%
	**Associated features**	Dilated duct	53 (22.75)	28 (23.93)	<0.001	65.4%
		No associated features	85 (36.48)	72 (61.54)		54.1%
		Skin changes (thickening, nipple retraction)	20 (8.58)	5 (4.27)		80%
		Pectoralis muscle invasion	2 (0.86)	0 (0.000)		100%
		Axillary adenopathy	65 (27.90)	6 (5.13)		91.5%
		Architectural distortion	8 (3.43)	6 (5.13)		57.1%
	**Maximum diameter**	<1 cm	23 (9.87)	48 (41.03)	<0.001	32.4%
		1–2 cm	54 (23.18)	34 (29.06)		64.3%
		≥2 cm	156 (66.95)	35 (29.92)		81.7%
**Mammography features**	**Density**	Low density	1 (0.43)	1 (0.86)	0.818	50%
		Equal density	188 (80.69)	96 (82.05)		66.2%
		High density	44 (18.88)	20 (17.09)		68.8%
					
	**Calcification distribution**	Suspicious	175 (75.11)	49 (41.88)	<0.001	78.1%
		No/alone	58 (24.89)	68 (58.12)		46.0%
	**Calcification morphology**	Suspicious	177 (75.97)	45 (38.46)	<0.001	79.3%
		No/benign coarse calcification	56 (24.03)	72 (61.54)		43.8%
**Ultrasound features**	**Echo pattern**	Hypoechoic/heterogeneous	216 (92.70)	103 (88.03)	0.147	67.8%
		Isoechoic/hyperechoic	17 (7.30)	14 (11.97)		54.8%
	**Posterior features**	No posterior features	146 (62.66)	98 (83.76)	<0.001	59.8%
		Shadowing	87 (37.34)	19 (16.24)		82.1%
	**CDFI peripheral vascularity**	Poor	43 (18.46)	61 (52.14)	<0.001	41.3%
		Rich	190 (81.55)	56 (47.86)		77.2%
	**Strain elasticity (5 points**)	2 points	69 (29.61)	66 (56.41)	<0.001	51.1%
		3 points	126 (54.08)	38 (32.48)		76.9%
		4 points	35 (15.02)	13 (11.11)		72.9%
		5 points	3 (1.29)	0 (0.00)		100%

Unless otherwise indicated, the value represents the number of patients, with the percentage shown in parentheses.

BPE, background parenchymal enhancement; FGT, amount of fibroglandular tissue; TIC, time signal intensity curve.

### 3.2 Selection of parameters by LASSO logistic regression

**Figure 2:**
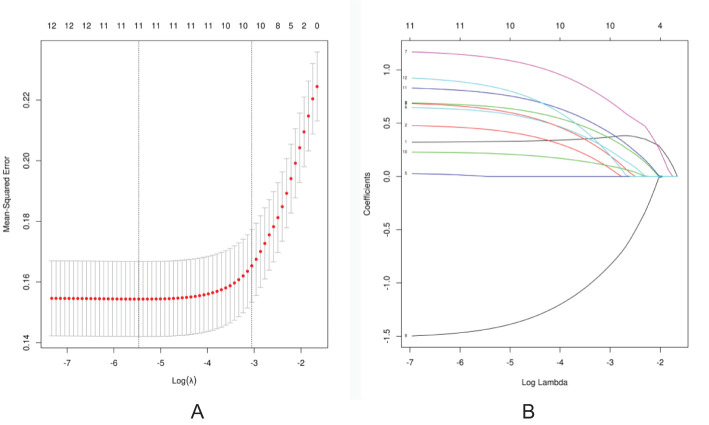
Screening of discriminative imaging and clinical indicators using the LASSO regression algorithm. **(A)** The 10-fold cross-validation method is used in the LASSO model to screen out the feature set with the best performance. The vertical dotted line represents the log(*λ*) value corresponding to the optimal input value. A total of 10 features were selected in this study. **(B)** Coefficient convergence graph of imaging indicators and clinical features using 10-fold cross-validation in the LASSO model; each curve in the graph represents the trajectory of each .

The LASSO regression method was used to select the indicators to avoid overfitting and to solve the problem of serious collinearity (**Figure 2**). In this study, the standard error *λ* of the minimum distance was selected as 0.047, and the selected variables of the corresponding model were as follows: age, ADC value, calcification distribution, calcification morphology, peripheral vascular supply displayed by CDFI, maximum diameter of the lesion, NME internal enhancement pattern, NME distribution, TIC curve, and clinical palpation hardness.

### 3.3 Construction and verification of the nomogram

**Figure 3:**
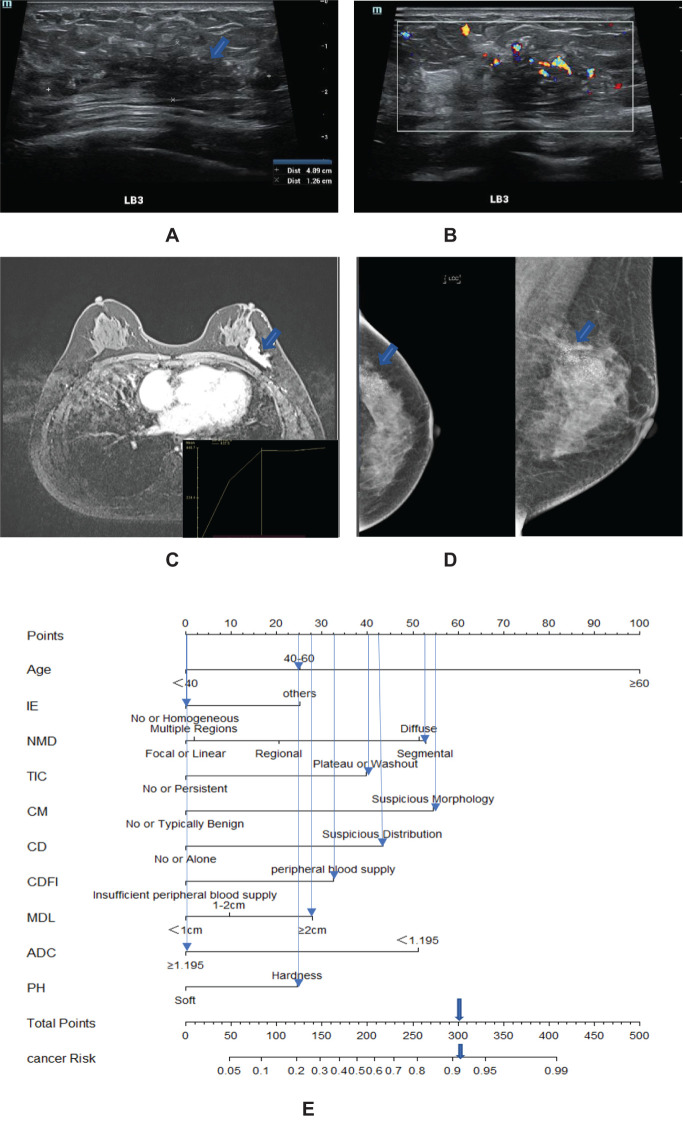
A 57-year-old woman with high-grade ductal carcinoma *in situ* at 3 o’clock on the left breast (blue arrow). **(A)** The maximum diameter of the lesion is 4.89 cm. **(B)** Ultrasound CDFI showed a rich blood supply around the lesions. **(C)** MRI showed a segmental distribution, and the time signal intensity curve showed a plateau shape. **(D)** Mammography showed fine pleomorphic calcifications with segmental distribution. **(E)** Adding the scores assigned by each covariate to get a total score of 300 points, the probability of risk cancer is 90%–95%, and it is classified as BI-RADS 4C. IE, internal enhancement; NMD, non-mass distribution; CD, calcification distribution; CM, calcification morphology; MDL, the maximum diameter of the lesion; PH, palpation hardness.

**Figure 4:**
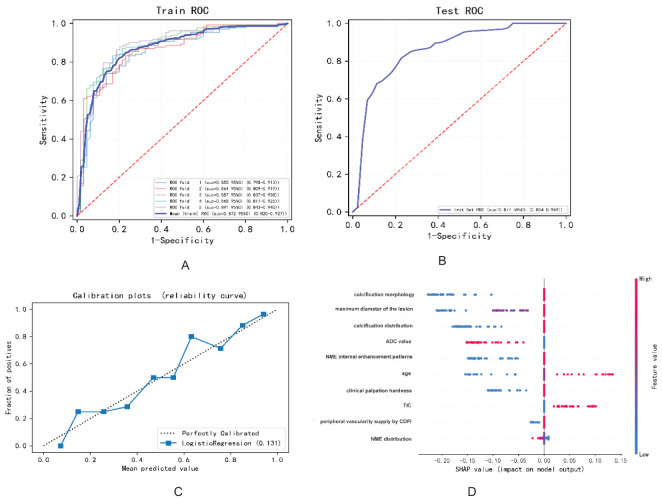
The diagnostic performance of the model in predicting benign and malignant breast NMLs: **(A)** ROC curve of the training set; **(B)** ROC curve of the test set; **(C)** model calibration curve graph; **(D)** variable contribution graph.

Based on the final screened variables, 10 indicators were finally used to construct a nomogram to predict the likelihood of malignancy in NMLs (**Figure 3**). The model assigns the score according to the weight of the covariates, then a straight line is drawn upward, to the point of the axis at the top to obtain the score based on each covariate. Total points are calculated by adding all the points obtained from each covariate. The final sum was placed on the total score axis, and a straight line was drawn downward from there to obtain the probability of cancer risk. The pathological results were used as the “gold standard.” The test set consisting of *N* = 105 cases (30.00%) was randomly selected from the overall sample, the remaining samples were used as the training set for five-fold cross-validation (**Figures 4A, B**), and the areas under the ROC curves (AUCs) of the training set and the test set were compared. The training set AUCs were 0.873 (0.820–0.927), and the test set AUCs were 0.877 (0.804–0.949). The model calibration curve showed floating around the baseline, indicating that the model is a good fit (**Figure 4C**). The model results were interpreted by calculating the contribution of each feature to the predicted results (**Figure 4D**).

Utilizing the final set of screened variables, we constructed a nomogram with 10 indicators to predict the probability of malignancy for NMLs, as illustrated in **Figure 3**. The model assigns scores to each covariate based on its weight. Users draw a straight line upward from each covariate’s score to the top axis to determine the points for each factor. The total score is calculated by summing the points of all the covariates. This total is then placed on the total score axis, and a straight line is drawn downward to ascertain the individual’s probability of developing cancer. Pathological outcomes served as the definitive “gold standard” for comparison.

### 3.4 BI-RADS classification of NMLs based on the nomogram predicting the malignant probability

The total score was obtained by adding the scores corresponding to each index, and the vertical line was drawn downward to obtain the corresponding malignancy probability. Finally, a total score of 0 was classified as category 2; a total score of fewer than 25 points was classified as category 3; 190 points ≤ total score < 330 points were classified as category 4C; and a total score ≥330 points was classified as category 5. The positive predictive values of the original BI-RADS category and the new BI-RADS category were obtained (**Table 3**).

**Table 3:** Original BI-RADS category and model prediction BI-RADS category.

		Benign, *N* (%)	Malignant, *N* (%)	Total	*p*-value
**Original BI-RADS category**	**3**	1 (100.00)	0 (0.00)	11	*p* < 0.01
	**4A**	15 (96.42)	11 (13.58)	81	
	**4B**	65 (37.80)	51 (62.20)	82	
	**4C**	32 (7.45)	87 (92.55)	94	
	**5**	0 (0.00)	82 (100.00)	82	
**Model prediction BI-RADS category**	**2**	3 (100.00)	0 (0.00)	3	*p* < 0.01
	**3**	1 (100.00)	0 (0.00)	1	
	**4A**	15 (93.75)	1 (6.25)	16	
	**4B**	65 (73.87)	23 (26.13)	88	
	**4C**	32 (19.16)	135 (80.84)	167	
	**5**	2 (2.67)	73 (97.33)	75	

Unless otherwise indicated, the value represents the number of patients, with the percentage shown in parentheses.

BI-RADS, Breast Imaging Reporting and Data System.

## 4 Discussion

Breast MRI is acknowledged for its superior sensitivity in women with an increased risk of breast cancer, and its use in screening programs is steadily growing (15). Particularly for NMLs, MRI stands out as the most sensitive imaging modality. Dynamic contrast-enhanced magnetic resonance imaging (DCE-MRI) enables a thorough and systematic evaluation of breast lesions, including the assessment of vascular characteristics. NME can exhibit intense enhancement, which helps to make the lesions more conspicuous (16, 17). While MRI studies have reported high sensitivity rates of approximately 90% or higher, they are also associated with elevated false-positive rates (4, 18, 19). In mammography, the detection of NMLs is contingent upon variations in glandular density or the distribution of microcalcifications, given the lack of a space-occupying effect. Asian women, who often have denser breast tissue, may experience more false negatives in mammography. A prospective multicenter study has confirmed the diagnostic value of contrast-enhanced MRI in the decision-making process for breast lesions with microcalcifications. The sensitivity, specificity, PPV, and NPV of DCE-MRI for detecting suspicious microcalcifications on mammography are 95.2%, 40.2%, 49.2%, and 93.3%, respectively (20). The synergistic use of ultrasound and mammography has been shown to markedly enhance the diagnostic accuracy of breast lesions (21–23). On mammography, NMLs can present as calcifications, asymmetries, or architectural distortions. Asymmetry on mammograms, stemming from increased glandular density, appears as a local echo that differs from the surrounding tissue on ultrasound. Architectural distortion is often visualized as an entanglement of Cooper’s ligaments on ultrasound. While mammography excels at identifying calcifications, the combined use of ultrasound and mammography significantly improves the diagnostic yield of calcifications in breast lesions (24). If a lesion is clearly identified as a mass by any of the imaging modalities, it is classified as a mass lesion; otherwise, it is categorized as an NML. This approach also compensates for missed diagnoses of NMLs that may occur due to glandular overlap on mammograms. On ultrasound, NMLs can manifest as hypoechoic areas or regions with blurred, altered echogenic texture, which can be difficult to differentiate from fat echoes. The use of color Doppler has been shown to increase the specificity of ultrasound in identifying malignant NMLs in the breast without reducing sensitivity (25).

Malignant lesions, including ductal carcinoma *in situ* (DCIS), invasive ductal carcinoma (IDC), and invasive lobular carcinoma (ILC), can present as NMLs, whereas benign conditions such as fibrocystic changes, breast adenosis, intraductal papilloma, and inflammatory processes can also exhibit non-mass-like enhancement (26, 27). The majority of NMLs are characterized by their complex histopathological composition (28). In our study, the most prevalent malignant histopathological type was DCIS (36.91%), followed by mixed carcinoma (19.31%). Within the benign group, adenopathy or fibrocystic degeneration was the most common, representing 48.72% of the cases. These findings are corroborated by Bartels et al. (27), who also noted a predominance of DCIS and a majority of benign NMLs manifesting as adenopathy or fibrocystic degeneration. Our analysis of the imaging features of NMLs disclosed a significant overlap between malignant and benign presentations, with NME being more frequently associated with malignancy according to the BI-RADS classification, aligning with our findings. Importantly, NME has been linked to an increased rate of false positives in breast MRI and a consequent rise in unnecessary biopsies, as noted in some studies (3).

LASSO regression, a technique for variable selection in high-dimensional generalized linear models, was employed in this study to identify significant indicators through LASSO logistic regression. Based on the contribution degree (the size of the regression coefficients) of various influencing factors in the model to the outcome variable, scores were assigned to each level of the influencing factors. The total score was obtained by summing up the scores of all the influencing factors. Finally, the predicted value of the outcome event for that individual was calculated from the functional transformation relationship between the total score and the probability of the outcome event occurring. Nomograms are often used to predict disease risks and to assess patient conditions and other aspects. The variables selected included calcification morphology, distribution, clinical palpation hardness, ADC value, TIC, maximum diameter of the lesion, internal enhancement patterns and distribution of NME, peripheral vascularity as indicated by CDFI, and age. This approach allowed us to develop a more refined model by employing a penalty function that reduced the number of variables and effectively mitigated overfitting. To validate the clinical utility of the nomogram, patients were randomly assigned to either the training or test group. As depicted in **Figure 4**, our model demonstrated a significant ability to differentiate between benign and malignant lesions.

According to the variable contribution map depicted in **Figure 4D**, the three most important variables were calcification morphology, the maximum diameter of the lesion, and the distribution of calcifications. Amorphous or coarse heterogeneous calcifications are considered to be of intermediate concern, whereas fine pleomorphic, fine linear, or fine linear branching calcifications are associated with a higher probability of malignancy (29). In this study, the calcification distribution was categorized into a suspicious distribution group and a single or no calcification group. It is widely accepted that clustered calcifications are the most common and are associated with the lowest degree of malignancy, whereas segmental distribution is considered to have the highest degree of malignancy. When assessing the malignancy of calcifications, both morphology and distribution are considered, and the presence of microcalcifications in NMLs increases the probability of malignancy by 5.9 times compared to cases with only microcalcifications (30). Previous research has indicated that lesion size can predict tumor malignancy, with the positive predictive value rising significantly as lesion size increases (8, 31). In our study, for lesions with a maximum diameter of 2 cm or greater, the malignancy rate was 81.7%.

The TIC for NMLs is less effective than for mass lesions, with malignant NMLs typically not exhibiting the typical washout patterns (32). Consequently, the diagnostic value of the TIC as a predictive index in NMLs is somewhat limited. In this study, the weight of the TIC ranked behind other parameters in the final set of 10 variables. The authors categorized the TIC curve into enhancement/persistent and plateau/washout groups, which may enhance the sensitivity of this indicator to some extent. Some studies have reported high diagnostic efficacy of ADC values in NME, with a sensitivity of up to 96% and a specificity of 100% (33–37). Conversely, other studies have suggested that the application of ADC values in non-mass lesions is limited (38). The diagnostic accuracy of ADC measurements is significantly influenced by lesion size and the choice and placement of the region of interest (ROI). In our study, lesions larger than 2 cm accounted for 54.6% of the total (191 out of 350), which may explain why the ADC value was retained.

Continuous variables were converted to categorical variables to facilitate clinical interpretation and the construction of the final nomogram. The optimal cutoff value was determined using the Youden index (with an ADC value of 1.195 × 10^−3^ mm^2^/s). Patients were stratified into three age groups in accordance with the United States Cancer Screening Guidelines (39). The nomogram can be utilized by assigning scores to each predictive factor, summing these scores to obtain a total score, and then using this total to determine the predicted risk. Pathological diagnoses served as the gold standard for comparison. The BI-RADS classification was applied to NML patients by stratifying cancer risk into BI-RADS categories (as shown in **Figure 3**), with the results presented in **Table 3**. The positive predictive value for each category was found to be within the malignancy range recommended by the BI-RADS lexicon.

The objective of this study was to develop a practical classification system for breast NMLs that leveraged the synergistic benefits of three principal imaging modalities: ultrasound, mammography, and MRI. By integrating repetitive indicators and capitalizing on the intuitive nature of visual nomograms, we aimed to predict the risk of malignancy across various BI-RADS categories. Our initial application of the BI-RADS classification revealed a marginally elevated positive predictive value in comparison to reference values for categories 4A and 4B. The revised classification demonstrated a notable increase in the proportion of category 4C lesions, with positive predictive values aligning within the malignancy range as per the BI-RADS lexicon.

This study addressed the challenge of underestimation by experienced radiologists and specialized breast researchers, and overestimation by community clinics and primary care centers. By introducing a nomogram, we proposed an objective method for obtaining non-mass BI-RADS classifications, thereby reducing interradiologist diagnostic variability and enhancing clinical applicability.

In conclusion, our study demonstrated the potential of a risk-predictive BI-RADS stratification model for non-mass breast lesions, leveraging a combination of clinical and imaging parameters. The model developed demonstrated good performance in both the training and testing sets, with high AUC values and positive predictive values for different BI-RADS categories. Nomograms not only assist in assessing the patient’s condition but also help doctors explain the severity and risk of disease to patients. Across various fields, nomograms provide a rapid, concise, and user-friendly calculation tool that contributes to improving work efficiency and accuracy. In summary, nomograms are powerful tools that transform complex mathematical relationships into intuitive and easily understandable graphs, providing strong support for research and practice in various fields. However, it is important to acknowledge the limitations of this study, such as the relatively small sample size and the need for further validation in larger and more diverse populations.

Future extensions of this study could include the incorporation of additional imaging modalities or biomarkers to improve the accuracy of risk stratification for non-mass breast lesions. Furthermore, prospective studies are needed to assess the clinical utility of this new risk prediction model in guiding treatment decisions and improving patient outcomes. Overall, this study opens up avenues for further research and development in the field of breast imaging and risk assessment.

## Data Availability

The original contributions presented in the study are included in the article/supplementary material. Further inquiries can be directed to the corresponding author.
